# Effects of water exchange rate on morphological and physiological characteristics of two submerged macrophytes from Erhai Lake

**DOI:** 10.1002/ece3.4703

**Published:** 2018-11-20

**Authors:** Duan‐yang Yuan, Xianghuai Meng, Chang‐qun Duan, Zhi‐Hong Wei, Wei Gao, Jun‐jun Chang, Xing‐ju Lv, Ying Pan

**Affiliations:** ^1^ Yunnan Key Laboratory for Plateau Mountain Ecology and Restoration of Degraded Environments, School of Ecology and Environmental Sciences Yunnan University Kunming China; ^2^ Research Center of Erhai Lake Dali China; ^3^ Department of Biology Nanjing University Nanjing China

**Keywords:** hydrological variation, morphological plasticity, plant biomass, plant nutrient, submerged plant

## Abstract

Growth patterns of aquatic macrophytes have been shown to vary in response to hydrological properties; however, such properties are typically characterized by water level fluctuation, flow velocity, flooding season, and sedimentation, but not by water exchange rate (WER). Herein, we experimentally investigated how WER (three levels: exchange 0%, 20%, and 40% of total water per day) affects water and sediment properties, and the consequences that these variations have on the individual responses of two submerged macrophytes, *Hydrilla verticillata* and *Myriophyllum aquaticum* which were planted in two different sediment types (sand and clay). In the experiment without ramets, it was found that turbidity, pH value, and dissolved carbon dioxide concentration of the system water were statistically unaffected by WER, while water dissolved oxygen (DO) concentration and sediment oxidation–reduction potential (ORP, in both sediments) consistently increased with increasing WER, regardless of experimental time. In the experiment containing ramets, biomass accumulation and relative growth rate (RGR) of both species gradually increased with increasing WER regardless of sediment type. The mechanisms were related to (a) increased oxygen availability, as indicated by gradually increased water DO concentration and sediment ORP; and (b) enhanced phosphorus (P) and nitrogen (N) absorbing abilities associated with stimulated root growth, reflected in increased mean root length, specific root length, and the root/above‐ground biomass ratio, with increasing WER. Additionally, in the experiments containing ramets, significant linear relationships were consistently detected between sediment ORP and root parameters, root parameters and plant nutrients (N and P), and plant nutrients and plant growth conditions (biomass accumulation and RGR). These results demonstrate that WER plays an important role in determining oxygen availability and thus impacts the growth of submerged macrophytes by altering the ability of roots to absorb nutrients, indicating that ecosystem functions are more sensitive to WER than previously recognized.

## 1 INTRODUCTION

Hydrological variation plays a critical role in determining ecological processes in aquatic environments (Maltchik, Rolon, & Schott, 2007). Hydrological elements are diverse and complex and include features such as runoff amount, water level fluctuation, sedimentation, and flooding season (Bao et al., 2012). Of these, the amount of runoff has ecological consequences that can assume two different forms: as flow velocity in running water (e.g., rivers and channels), where turbulence at the water–air surface is strong; and as water exchange rate (WER) in relatively stagnant water (e.g., lakes and ponds), where turbulence at the water–air surface is weak. Extensive research has been conducted on the ecological functions of flow velocity (e.g., Janauer, Schmidt‐Mumm, & Schmidt, 2010; Hintz & Wellnitz, 2013); however, few studies have focused on WER.

Variations in WER can have numerous effects on water and sediment environments, including the dissolved oxygen (DO) concentration, dissolved carbon dioxide (DCD) concentration, and turbidity of waterbodies, as well as the nutrient and redox status of both waterbodies and sediments, and thus may have profound effect on aquatic organisms. For example, in lakes and ponds, the reduction in WER prevents the replenishment of DO from the incoming water and inhibits oxygen transfer across the air–water and water–sediment interfaces (Chatelain & Guizien, 2010; Jirka, Herlina, & Niepelt, 2010). In addition, low WERs could lead to the accumulation of metabolites (ammonia, nitrite, DCD, etc.) from aquatic animals and microorganisms (Hopkins, Hamilton, Sandier, Browdy, & Stokes, 1993). Collectively, a reduction in WER may inhibit the growth of many aquatic animals, such as various species of fish (Good et al., 2009) and shrimp (Hopkins et al., 1993; Lemonnier, Jlm, Brizard, & Herlin, 2003), due to oxygen deficiency and physiological changes (e.g., appetite suppression). However, few studies have extensively examined the influence of WER on the individual performance of aquatic plants (Madsen, Chambers, James, Koch, & Westlake, 2001). Such investigations are essential to assess their current status and to predict possible successional changes in aquatic plant communities (Schutten & Davy, 2000).

Individual characteristics of aquatic plants (here refer mainly to emergent and floating‐leaved macrophytes) may vary with WER due to changes in environmental conditions. First, low WERs have been shown to create anoxic environments for aquatic plants growing below the water surface. Aquatic plants can acclimate to this stress by developing short and thick roots, reducing the root mass ratio and decreasing specific root length (SRL, the length‐to‐mass ratio of a root fragment). These adjustments allow plants to acclimate to oxygen‐deficient conditions by facilitating the aeration of root tissues relies on the formation of aerenchyma tissues, and/or by reducing root oxygen demand (Li, Xie, Ren, Luo, & Huang, 2007; Pan, Xie, Chen, & Li, 2012). However, the inhibition of root growth and elongation may hinder nutrient acquisition, especially phosphorus (P) and nitrogen (N) (Pan, Xie, Deng, Tang, & Pan, 2014; Xie, An, Yao, Xiao, & Zhang, 2005). Therefore, a trade‐off must occur between oxygen supply and nutrient acquisition, that is, the ability of an aquatic plant to adapt to oxygen‐deficient conditions gradually declines with decreasing WERs in order to meet the plant's increasing need for nutrients. In contrast, extremely high WERs result in mechanical disturbance and sediment resuspension, which can further reduce rooting and light absorption abilities, and thereby impact the survival and growth of aquatic macrophytes (Henry & Myrhaug, 2013; Madsen et al., 2001). In brief, the influence of WER in determining individual characteristics of aquatic macrophytes should, therefore, not be neglected.

Compared with other aquatic macrophytes, submerged macrophytes are likely to be more sensitive to WER variation because they cannot shift most parts of the plant body above the water surface to improve access to oxygen and carbon dioxide. Although submerged macrophytes are well known for their ability to produce oxygen, especially the release of substantial amounts from the roots to the sediment during illumination (Sand‐Jensen, Prahl, & Stokholm, 1982), these plants also face the challenges of oxygen deficiency. This is due to the fact that submerged macrophytes basically could not produce oxygen under low‐light conditions, such as during nighttime or in water with low transparency (Li et al., 2007; Xie, Luo, Ren, & Li, 2007). In addition, previous studies have found that the growth and primary production of submerged macrophytes can be limited by low concentrations of DCD (Demars & Trémolières, 2009; Hussner, Mettler‐Altmann, Weber, & Sand‐Jensen, 2016). In brief, individual characteristics of submerged macrophytes may be greatly affected by WER due to variation in both DO and DCD concentrations; however, these effects had not yet been investigated.

The aim of this study is to determine the impacts of WER on water (turbidity, pH value, and DCD and DO concentrations) and sediment properties (ORP) and then to assess the consequences on biomass accumulation, relative growth rate (RGR), and morphological (including root/above‐ground biomass ratio, mean root length, root diameter, and SRL) and physiological (plant N and P concentrations) characteristics of two submerged macrophytes *Hydrilla verticillata* and *Myriophyllum aquaticum*. These two species were selected because they are commonly found in many shallow lakes throughout southwest China, including Erhai Lake (Li et al., 2015); besides, their morphological and physiological traits have previously been reported to be sensitive to hydrological variation (Hussner, Meyer, & Busch, 2009; Kennedy, Horth, & Carr, 2009; Wersal, Cheshier, Madsen, & Gerard, 2011). The treatments consisted of three levels of WER (exchange 0%, 20%, and 40% of total water per day) and two types of sediment (clay and sand). We hypothesized that along with increasing WER: (a) water DO concentration and sediment ORP would gradually increase, and as a result, roots would become longer and thinner, and more biomass would be allocated to the roots due to ease the stress of oxygen deficiency, (b) which would further promote the P‐ and N‐absorbing abilities, and (c) all these changes combined with potentially increased water DCD concentration will promote the growth of both species.

## 2 METHODS

### 2.1 Plant materials

In May, 2017, *Hydrilla verticillata* and *Myriophyllum aquaticum* were collected from Erhai Lake of Chongyizhuang Village (25º38*'*39.2*''*N, 100º12*'*54.0*''*E), Yunan province, China. *H. verticillata* is an obligately submerged species, while *M. aquaticum* is an amphibious macrophyte with both submerged and floating leaves (Hussner et al., 2009; Kennedy et al, 2009; Wersal et al., 2011). We used two species with different life forms in order to reduce the species‐specific effects on our experimental results. After collection, sample plants of the two species were cultured in plastic buckets (67 ×  45 ×  55 cm) in an unheated plastic greenhouse on the grounds of Yunnan University. Buckets were prepared with 6 cm of clay (containing 5.49 g/kg organic matter, 2.24 g/kg exchangeable N, and 0.9 g/kg exchangeable P excavated from the same location where *M. aquaticum* was collected) and 20 cm (from the bottom of the bucket) of tap water. This early culture was allowed to grow for one month in order to minimize the potential effect of local environments. New ramets of *H. verticillata* (*n* > 100) of about 5 cm in height and apical shoots of *M. aquaticum* (*n* > 100) were then chosen for further culture in four other buckets under the same conditions as those mentioned above. Apical shoots of *M. aquaticum* were collected from multiple parent individuals of similar size cut into equal lengths of 5 cm following previous studies (e.g., Maberly & Madsen, 2002; Wersal & Madsen, 2011). This culture lasted for about 2 weeks to cultivate suitable experimental ramets.

### 2.2 Experimental design

The experiments were conducted in the same greenhouse and began on 15 July 2017. In total, 36 ramets per species of a similar size (length [mean ± *SD*,* n* = 36]: 9.03 ± 0.34 cm for *H. verticillata* and 11.24 ± 0.54 cm for *M. aquaticum*]) were used in the experiments. The fresh weights of all these ramets were individually measured. Meanwhile, five other ramets per species of similar size as experimental ones were dried at 85°C for 48 hr and reweighed to determine a wet‐to‐dry conversion factor. Therefore, the fresh weights of these 36 ramets could be individually converted to dry weight at the initial of the experiment. Experimental ramets were individually transplanted into PVC tubes (10 cm height × 14 cm diameter, bottoms enclosed with nylon netting). For each tube, four drainage holes (0.5 cm diameter) were drilled evenly 6 cm from the bottom, and 6 cm of corresponding sediment was added before migrating.

Two different types of sediment: clay and sand, representing the main sediment types encountered in Erhai lake (Chen, Wan, & Xu, 2000), were used in our experiments since it is generally recognized that the ecological consequences of hydrological variations are strongly associated with sediment types in freshwater ecosystems (Wang, Wang, Zhao, & Yang, 2012; Xie et al., 2005). The clay was the same as that used for the plant incubation, and the sand (containing 0.91 g/kg organic matter, 0.67 g/kg exchangeable N, and 0.11 g/kg exchangeable P) was also collected from Erhai Lake. Prior to the experiments, humus soil was thoroughly mixed into the clay and sand sediments at a mixture of 3:17 (v/v) of humus soil to corresponding sediment, so as to homogenize nutrient states between the two different sediments as much as possible. Initial analysis indicated that organic matter was indistinguishable between the two sediments; however, bulk density was significantly higher in the sand than in the clay sediment (Supporting Information Table S1).

Three water exchange treatments were designed in our experiment: motionless (0% exchange of total water per day, no replacement), medium WER (20% exchange, complete replacement in 5 days), and high WER (40% exchange, complete replacement in 2.5 days; Supporting Information Figure S1). Erhai Lake is a large rift lake with low WER, and >2 years are needed for a complete replacement; however, in some areas of the lake (e.g., inlets and outlets), WER could be much higher, and one replacement may take only a few hours (Wan et al., 2003; Zhang, Wang, & Wu, 2014). Therefore, the variable range of WER used in the present study is reasonable. Tap water (containing 160 µg/L NH^4+^‐N, 745 µg/L NO^3−^‐N and 130 µg/L PO_4_^3+^‐P) was first aerated with sterile filtered air (Midisart 2000 filters; Sartorius, Dandenong, Vic., Australia) in an aeration bottle (to homogenize DO and DCD) and then supplied as needed in each WER treatment throughout the experiment.

Collectively, a two‐way factorial design was applied that combined the three WERs and two sediment types, resulting in six treatments. The 72 tubes were placed into nine plastic buckets (90 ×  55 ×  61 cm, total volume about 300 L). Each WER treatment consisted of three buckets, with each bucket containing eight tubes (two tubes per sediment type per species). The tubes were placed randomly within each bucket. All plant leaves remained above the sediment surface during the experiment. All ramets were harvested before their leaves grew out of the water surface because submerged macrophytes can absorb oxygen and carbon dioxide directly from air by aerial leaves and hence could alter the individual plant performances in response to WER variation (here refer mainly to *M. aquaticum*). Furthermore, an additional experiment was conducted with the same design, viz., PVC tubes (four tubes per sediment type per bucket) and corresponding sediments for each of the above‐mentioned WER treatments (three buckets per treatment), but without ramets. This experiment was carried out before the experiment containing ramets in order to establish the most sensitive environmental factor associated with WER variation without the interference of submerged plants.

### 2.3 The experiment in the absence of ramets

This experiment had lasted for 11 days. During the experimental period, turbidity, pH value, and DCD and DO concentrations of the system water in each bucket were measured every two days at a depth of 56 cm (3 cm above tube bottom) during both daytime (6:00–21:00) and nighttime (21:00–6:00). The turbidity, pH value, DCD concentration, and DO concentration were determined using a turbidity meter (WGZ‐4000AP, Shanghai Xinrui Instruments Co. Ltd, Shanghai, China), a pH meter (Beckman Φ‐200, Beckman, Fullerton, CA, USA), a DCD meter (AMT‐SC200, Shengzheng Yunchuan Wulian Technologies Co. Ltd, Shengzheng, China), and a DO meter (HQ40d, Hach, Loveland, CO, USA), respectively. Additionally, sediment ORP (using one randomly chosen chamber per bucket per sediment type) was tested on Day 11 at the root site (3 cm above the bottom of the tube) using a platinum electrode with an Ag/AgCl reference electrode (BPH‐221; Bell Analytical Instruments, Co., China) inserted directly into the PVC tubes. Sediment ORP was measured at the end of this experiment in order to prevent its influence on water qualities.

### 2.4 The experiment in the presence of ramets

#### 2.4.1 Water and sediment qualities

In this experiment, DO concentration was measured in daytime (6:00–21:00) on days 10, 20, 30, 40, and 50 and in nighttime (21:00–6:00) on Day 50, while, sediment ORP (using five randomly chosen chambers per treatment per species) were measured on Day 51 in both daytime and nighttime, respectively, following the methods mentioned above.

#### 2.4.2 Plant morphological and physiological characteristics

All plants were harvested after sediment ORP measurement. At harvest, the entire root system of each plant was carefully excavated from the sediment and rinsed with tap water. Plants were divided into roots (including rhizomes), stems, and leaves, and the fresh weight of each plant division was recorded (±0.0001 g). Half of the fresh roots were used for root morphological analysis; the remaining roots and other plant tissues were dried at 85°C for 48 hr and reweighed to determine a wet‐to‐dry conversion factor, which was used to concert all sample fresh weights to dry weights. Dry weights were used in the calculations. Biomass accumulation was measured as the sum of the root, stem, and leaf mass, and the ratio of root/above‐ground biomass was defined as the ratio of root biomass to stem + leaf biomass. RGR = (In *w*_2_ – In *w*_1_)/(*t*_2_ **− *t*_1_), where *w*_1_ was the initial dry mass, *w*_2_ the dry mass at harvest time *t*_2_, and (*t*_2_ − *t*_1_) the experimental time.

At least three (it is difficult to find more than three roots in some plants) representative full‐grown adventitious roots (growing from rhizomes and below the sediment surface) were selected from each plant, and the mean root lengths and diameters were measured using a Vernier caliper and microscope equipped with an ocular micrometer (Olympus BX51; Olympus, Japan), respectively. The total root length and fresh weight of the three adventitious roots (cut off lateral roots) were recorded for each plant, and SRL was defined as total root length divided by fresh root mass. Values for mean root length, diameter, and SRL were first averaged for each plant and then for each treatment.

After harvest, plant parts were ground into powder and mixed together for measurement of plant nutrient concentrations. All samples were digested with H_2_SO_4_‐H_2_O_2_ and analyzed for plant P and N concentrations using colorimetric analysis (Shi, 1994). Each treatment was performed in triplicate.

### 2.5 Statistical analysis

All data were tested for normality and variance homogeneity prior to analyses. Data on biomass accumulation, the root/above‐ground biomass ratio and plant P concentration were log_10_‐transformed to achieve normality and homogeneity.

In the experiment without ramets, one‐way ANOVAs followed by the Tukey test were performed to test the effects of WER on turbidity, pH value, and DCD and DO concentrations of the bucket water and on ORP value of the system sediment for each observation time. Besides, one‐way repeated measures ANOVA followed by a Bonferroni post hoc test was used to test the effects of WEU on the measured variables of system water.

In the experiment containing ramets, two‐ and three‐way ANOVAs followed by the Tukey test were performed to determine the effects of WER and experimental time on water DO concentration and sediment ORP, and the effects of WER, sediment type, and plant species on sediment ORP and growth, root parameters, and plant nutrient contents of two submerged species, respectively. Additionally, linear regression analyses were conducted to determine the relationships between sediment ORP and root parameters, root parameters and plant nutrients, and plant nutrients and plant growth conditions. All statistical analyses were performed with SPSS 22.0 (SPSS Inc., USA).

## 3 RESULTS

### 3.1 Effects of WER on water and sediment qualities

#### 3.1.1 In the absence of ramets

In both daytime and nighttime, turbidity (Table 1; Supporting Information Table S2), pH value (Table 1; Supporting Information Table S3), and DCD concentration (Table 1; Supporting Information Table S4) of the bucket water were statistically unaffected by WER (*p* > 0.05); however, water DO concentration (*p* < 0.01, Table 1; Supporting Information Table S5) and sediment ORP (*p* < 0.05, Supporting Information Table S6) consistently increased with increasing WER, throughout the experiment. For all three WERs, sediment ORP was significantly higher in the clay than in the sand treatment, irrespective of experimental time (*p* < 0.05; Supporting Information Table S6).

**Table 1:** Results of one‐way repeated measures ANOVA (*F* and *p* values) showing the effects of water exchange rate (WER) on turbidity, pH value, dissolved oxygen (DO) concentration, and dissolved carbon dioxide (DCD) concentration in both daytime and nighttime during the 11‐day‐long experiment without ramets

	Time (*T*)	*df*	WER (*W*)	*df*	*T × W*	*df*
Turbidity in daytime	13.869**	4	0.833^ns^	2	0.447^ns^	8
Turbidity in nighttime	8.802*	4	1.359^ns^	2	0.300^ns^	8
pH value in daytime	0.077^ns^	4	0.852^ns^	2	0.320^ns^	8
pH value in nighttime	1.027^ns^	4	0.147^ns^	2	0.188^ns^	8
DO concentration in daytime	48.645***	4	19.629**	2	0.342^ns^	8
DO concentration in nighttime	25.029**	4	119.216***	2	1.134^ns^	8
DCD concentration in daytime	0.873^ns^	4	1.866^ns^	2	0.419^ns^	8
DCD concentration in nighttime	0.853^ns^	4	1.988^ns^	2	0.160^ns^	8

^ns^ *p* > 0.05;

a *p* < 0.05;

b *p < *0.01;

c *p* < 0.001.

#### 3.1.2 In the presence of ramets

Water DO concentration was significantly affected by WER through the experiment (Supporting Information Table S7) and was affected by both WER and experimental time on Day 50 (*p* < 0.001; Supporting Information Table S8). Specifically, during the experimental period, DO concentration consistently increased with increasing WER (*p* < 0.02; Supporting Information Figure S2 and Table S7), regardless of experimental time. Meanwhile, at the end of the experiment (on Day 50), DO concentration was significantly higher in daytime than in nighttime for all three WERs (*p* < 0.05).

**Figure 1:**
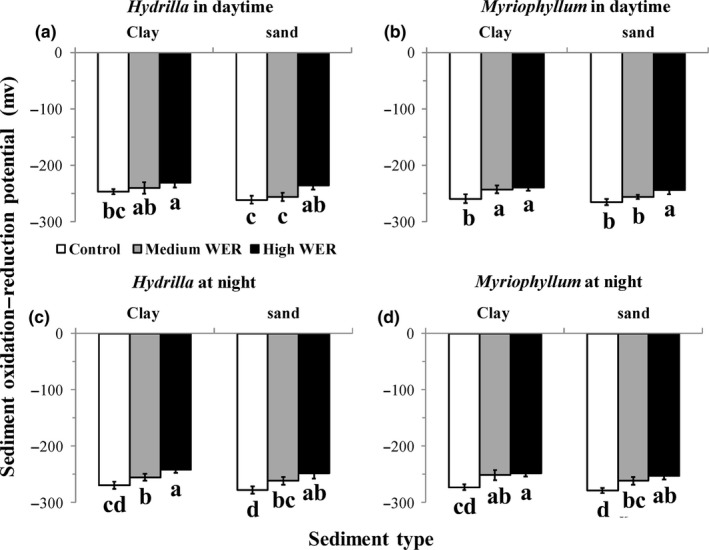
Sediment oxidation–reduction potential (mean ± 1 *SD*,* n* = 5) in chambers planted with *Hydrilla verticillata* (a, c) and* Myriophyllum aquaticum* (b, d) under three levels of water exchange rates and two types of sediment in daytime (a, b) and at night (c, d). Different letters indicate significant differences among treatments. Multiple comparisons of means were performed using the Tukey test at the 0.05 significance level

Sediment ORP was significantly affected by both WER and sediment type, regardless of experimental time (*p* < 0.001; Table 2; Supporting Information Table S8). Specifically, for both sediment types, sediment ORP consistently increased with increasing WER, irrespective of experimental time and plant–soil system (*p* < 0.05; Figure 1a–d). For all three WERs, sediment ORP was generally higher in the clay than in the corresponding sand treatment, which reaches significant levels in daytime on sediment ORP of *H. verticillata* in treatments with medium WER (Figure 1a) and on sediment ORP of *M. aquaticum* treated with medium WER (Figure 1b). Additionally, sediment ORP was generally higher in daytime than in nighttime at a given WER level and sediment type, irrespective of plant–soil system (Supporting Information Table S8).

**Table 2:** Summary of ANOVA results (*F* and *p* values) showing the effects of water exchange rate (WER), sediment type, and plant species on sediment oxidation–reduction potential (ORP; *n* = 5) in daytime and nighttime, as well as on plant biomass accumulation (*n = *6), relative growth rate (*n = *6), root/above‐ground biomass ratio (*n* = 6), mean root length (*n* = 6), root diameter (*n = *6), specific root length (SRL; *n = *6), and plant nitrogen (N; *n* = 3) and phosphorus (P; *n = *3) concentrations in *Hydrilla verticillata* and* Myriophyllum aquaticum*, respectively

	*df*	ORP in daytime (mv)	ORP in nighttime (mv)	Biomass accumulation (g dry wt/plant)	RGR (g g^−^^1^ dry wt day^−1^)	Root/above‐ground biomass ratio	Mean root length (cm)	Root diameter (µm)	SRL (m/g)	Plant *N* (mg/g)	Plant P (mg/g)
WER	2	44.847***	82.522***	51.108***	61.781***	14.235***	20.081***	19.127***	13.952***	147.407***	98.901***
Sediment type (*S*)	1	29.937***	16.354***	13.159**	16.653***	6.920*	30.139***	7.327**	12.836**	44.301***	68.587***
Plant species (*P*)	1	10.503**	1.278^ns^	125.048***	37.905***	0.584^ns^	2.999^ns^	27.634***	0.500^ns^	13.875**	90.005***
WER ×S	2	2.857^ns^	0.159^ns^	0.918^ns^	1.419^ns^	0.137^ns^	0.364^ns^	0.041^ns^	0.533^ns^	1.833^ns^	8.867**
WER ×P	2	1.623^ns^	1.623^ns^	3.084^ns^	2.713^ns^	0.396^ns^	0.179^ns^	0.874^ns^	0.132^ns^	17.587***	0.235^ns^
*S* × *P*	1	1.162^ns^	0.000^ns^	4.230*	5.471*	0.068^ns^	2.065^ns^	0.840^ns^	0.650^ns^	0.237^ns^	0.121^ns^
WER ×S* × P*	2	0.476^ns^	0.357^ns^	1.297^ns^	2.191^ns^	0.092^ns^	0.450^ns^	0.381^ns^	0.716^ns^	0.696^ns^	2.156^ns^

^ns^ *p* > 0.05;

a *p* < 0.05;

b *p < *0.01;

c *p* < 0.001.

### 3.2 Effects of WER on plant growth

#### 3.2.1 Biomass accumulation and RGR

**Figure 2:**
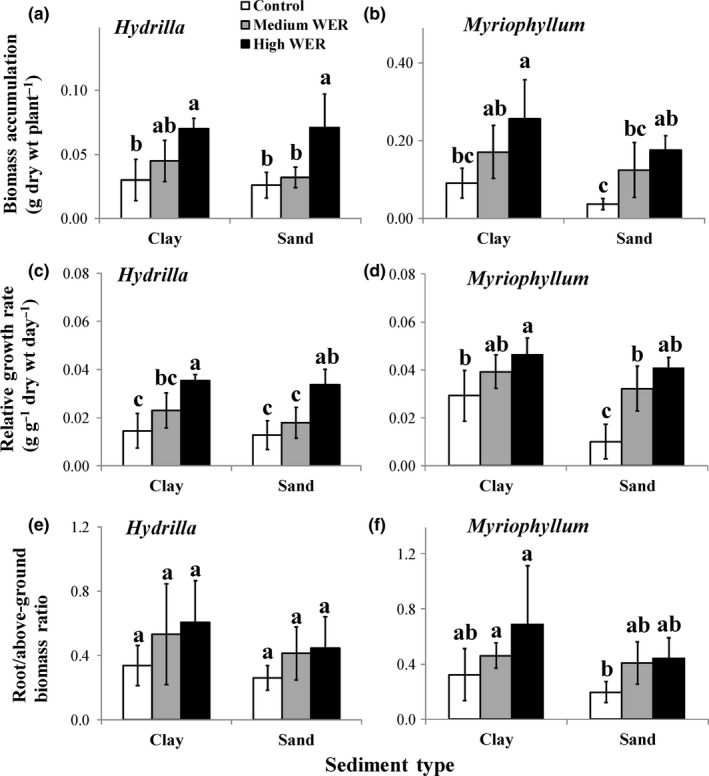
Variations (mean ± 1 *SD*,* n* = 6) in biomass accumulation (a, b), relative growth rate (c, d), and the ratio of root/above‐ground biomass (e, f) in *Hydrilla verticillata* (a, c, e) and* Myriophyllum aquaticum* (b, d, f) under three levels of water exchange rates and two types of sediments. Different letters indicate significant differences among treatments. Multiple comparisons of means were performed using the Tukey test at the 0.05 significance level

Biomass accumulation and RGR were significantly affected by both WER and sediment type (*p* < 0.01; Table 2). For both sediment types, biomass (Figure 2a,b) and RGR (Figure 2c,d) consistently increased with increasing WER, which showed significant differences between high WER treatments and controls, irrespective of plant species (*p* < 0.05). For all three WERs, biomass accumulation and RGR were generally higher in plants grown in the clay than in those grown in the sand sediment, irrespective of plant species.

#### 3.2.2 Root characteristics

**Figure 3:**
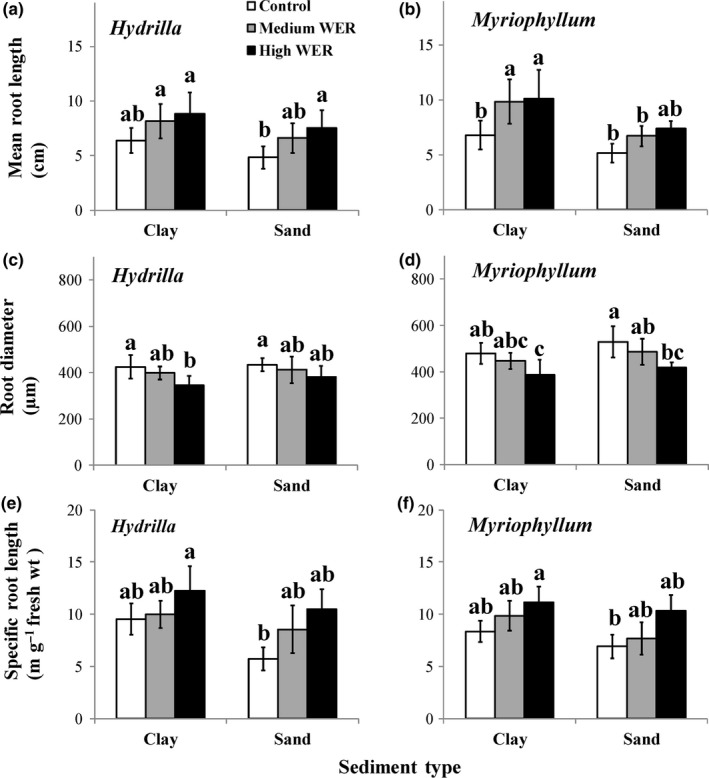
Variations (mean ± 1 *SD*,* n *= 6) in mean root length (a, b), root diameter (c, d) and specific root length (e, f) of *Hydrilla verticillata* (a, c, e) and* Myriophyllum aquaticum* (b, d, f) under three levels of water exchange rates and two types of sediment. Different letters indicate significant differences among treatments. Multiple comparisons of means were performed using the Tukey test at the 0.05 significance level

The root/above‐ground biomass ratio, mean root length, SRL, and root diameter were all significantly affected by both WER and sediment type (*p* < 0.05; Table 2). For both sediment types, the root/above‐ground biomass ratio (Figure 2e,f), mean root length (Figure 3a,b), and SRL (Figure 3e,f) exhibited increasing trends, while root diameter (Figure 3c,d) exhibited a decreasing trend with increasing WER in both species. In particular, WER had significant effects (*p* < 0.05) on mean root length of *H. verticillata* grown in sand (Figure 3a) and of *M. aquaticum* grown in clay (Figure 3b) and on root diameter of *H. verticillata* grown in clay (Figure 3c) and of *M. aquaticum* grown in both sediment types (Figure 3d). For all three WERs, root/above‐ground biomass ratio, mean root length, and SRL were generally higher, but root diameter was generally lower, in plants grown in the clay than those in the corresponding sand treatment, irrespective of plant species.

Furthermore, significant linear relationships were detected between sediment ORP and root parameters (from Supporting Information Figure S3–S6). Specifically, sediment ORP was significantly and positively associated with root/above‐ground biomass ratio (*R* ^2^ > 0.70; *p* < 0.04; Supporting Information Figure S3), mean root length (*R* ^2^ > 0.75; *p < *0.03; Supporting Information Figure S4), and SRL (*R* ^2^ > 0.79; *p < *0.02; Supporting Information Figure S6), but negatively associated with root diameter (*R* ^2^ > 0.75; *p < *0.03; Supporting Information Figure S5), regardless of experimental time and plant–soil system.

#### 3.2.3 Plant N and P concentrations

**Figure 4:**
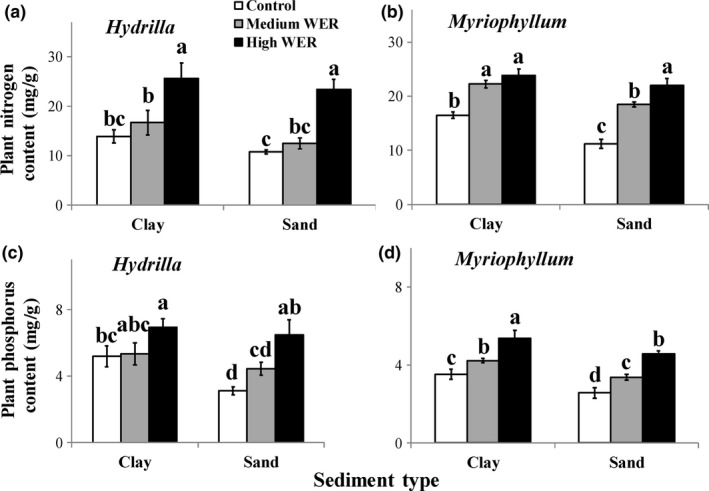
Variations (mean ± 1 *SD*,* n* = 6) in nitrogen (a, b) and phosphorus (c, d) concentrations in *Hydrilla verticillata* (a, c) and* Myriophyllum aquaticum* (b, d) under three levels of water exchange rates and two types of sediment. Different letters indicate significant differences among treatments. Multiple comparisons of means were performed using the Tukey test at the 0.05 significance level

Plant nutrients were significantly affected by WER and sediment type (*p* < 0.001; Table 2). For both sediment types, concentrations of plant N and P consistently increased with increasing WER in both species (*p* < 0.05; Figure 4a‐d). For all three WERs, concentrations of plant N and P were generally higher in plants grown in the clay than in those grown in the corresponding sand treatment in both species, which showed statistically significant differences (*p* < 0.05) in N concentrations of *M. aquaticum* treated with motionless or medium WER (Figure 4b), as well as in P concentrations of *H. verticillata* treated with motionless WER (Figure 4c) and of *M. aquaticum* regardless of WERs (Figure 4d).

Additionally, significant linear relationships were also detected between plant nutrients and root parameters (Supporting Information Figures S7 and S8). Specifically, concentrations of plant N and plant P were significantly and positively associated with root/above‐ground biomass ratio, mean root length, and SRL and negatively associated with root diameter in both species, respectively (*R* ^2^ > 0.60; *p* ≤ 0.05), with the exception of the insignificant relationship between plant N concentration and the root/above‐ground biomass ratio of *H. verticillata* (*R* ^2^ = 0.62; *p* = 0.064). Additionally, plant nutrients were generally positively associated with both biomass accumulation and RGR, regardless of plant species (*R* ^2^ > 0.80; *p* < 0.05; Supporting Information Figure S9).

## 4 DISCUSSION

Over the past decades, global climate anomalies and advancements in hydraulic engineering have resulted in greater uncertainties in the long‐term trends and regional differences of WER (Jacques, Sauchyn, & Zhao, 2010; Piao et al., 2010), which may have severe impacts on aquatic macrophytes (Pan, Zhang, Li, & Xie, 2016). The aim of this study was to identify the exact effects of WER variation on the individual characteristics of two submerged macrophytes, *H. verticillata* and *M. aquaticum*. Our results showed that the growth, morphology, and physiology of both species varied with WER, findings that are consistent with those of previous studies involving other aquatic organisms (e.g., aquatic animals and wetland plants; Lemonnier et al., 2003; Good et al., 2009). These results indicate that WER is an important feature of hydrological characteristics that should be considered when investigating the effects of hydrological variation on the structure and function of aquatic ecosystems.

### 4.1 Effects of WER on water and sediment qualities

In the experiment without ramets, water DO concentration gradually increased with increasing WER. DO concentration is very important to the functioning of the aquatic ecosystem, which is otherwise also influenced by many other factors. For example, reaeration at the water surface (controlled by turbulence and molecular diffusion; Jirka et al., 2010), replenishment from incoming water, and production by phytoplankton and aquatic macrophytes are potential contributors of oxygen (Chatelain & Guizien, 2010). In contrast, the respiration of aquatic organisms and the mineralization of organic matter could lead to a decline in DO concentration (Ayi et al., 2016; Chang, Ma, Chen, Lu, & Wang, 2017; Chapelle et al., 2000 **)**. In this experiment, the increased flow turbulence and thus oxygen transfer rate, and accelerated replenishment of oxygen from the incoming water were the main factors that contributed to the increase in water DO concentration. This is particularly true given that this experiment was carried out in the absence of aquatic animals, macrophytes and algae, and besides, organic matter content showed no significant difference across all treatment sediments prior to the start of the experiment, which could exclude the impact of other organic and inorganic factors on water DO concentration. Subsequently, continuously increased water DO concentration inevitably led to an increase in sediment ORP along with increasing WER (Pan et al., 2014).

Surprisingly, water DCD remained constant at high concentration and did not vary with WER, which may be related to equal organic matter content among experimental sediments. It is commonly understood that the destruction of organic matter can produce a large amount of carbon dioxide that might exceed the impact of WER variation on water DCD concentration in the present experiment (Marotta et al., 2010). Additionally, neither turbidity nor pH value of the system water was affected by the designed treatments. Collectively, we can conclude that oxygen availability was the most sensitive factor associated with WER variation in this experiment.

In the experiment containing ramets, water DO concentration and sediment ORP also increased with increasing WER, regardless of experimental time, further demonstrating the sensitivity of oxygen availability to WER variation. In addition, both water DO concentration and sediment ORP were significantly higher in the daytime than in the nighttime, indicating that the submerged macrophytes were releasing oxygen into the anoxic environments during illumination, consistent with the findings of many previous studies (e.g., Kemp & Murray, 1986). However, during the nighttime, the submerged macrophytes may still have to endure long‐term oxygen deficiency, as indicated by low values of water DO (between 2.41 and 3.71; Supporting Information Figure S2) and sediment ORP (<−240 across treatments; Figure 1) under these circumstances, which may have great impacts on plant performances.

### 4.2 Effects of WER on plant morphological and physiological characteristics

Maintaining a relatively high rate of oxygen uptake and minimizing the damage caused by anoxic stress are critical for submerged macrophytes that encounter anoxic conditions. Reducing the root mass ratio and developing shorter and thick roots are key morphological adjustments that submerged macrophytes often resort to in response to anaerobic stress, which aids in reducing radial oxygen loss and improving the oxygen transport efficiency from shoots to roots (Chen, Qualls, & Blank, 2005; Rascio, 2002; Xie et al., 2007). In response to increasing WER in the present study, root/above‐ground biomass ratio, mean root length, and SRL consistently increased but root diameter decreased in both species, which may be attributed to increased oxygen availability under such conditions, as was evidenced by the significant relationships between these variables and sediment ORP (see Supporting Information Figures S3–S6). These results support our first prediction, concerning the root morphological responses of the two species to increasing WER.

Root uptake is the primary pathway for nutrients (e.g., N and P) in submerged macrophytes (Madsen & Cedergreen, 2010; Xie et al., 2005). Therefore, easing oxygen deficiency stress will subsequently improve the ability of aquatic plants to absorb nutrients by shifting the trade‐off between oxygen supply and nutrient acquisition (Pan, 2012; Xie et al., 2007). As expected, increasing WER reduced oxygen deficiency stress in both species, allowing their plants to allocate more resources and energy toward growth and elongation of roots, which subsequently increases plant N and P concentrations. These deductions have been shown by the significant and positive relationships between the root/above‐ground biomass ratio, mean root length, and SRL with plant N and P contents (see Supporting Information Figures S7 and S8), respectively. These findings provide support for our hypothesis 2. Improving plant nutrient status ultimately resulted in higher biomass accumulation and RGR in both species at the end of experiment, reflected in the significant and positive relationships between sediment nutrients and plant growth conditions (including biomass accumulation and RGR, Supporting Information Figure S9), which is consistent with our hypothesis 3.

Additionally, we have clearly shown that sediment type also exerted an important role in modulating plant performances. Compared with clay sediment, sand sediment had significantly higher bulk density (Supporting Information Table S1) and thus lower porosity (indicated by relatively low ORP value; Figure 1). As a result, roots were shorter and thicker, and nutrient absorption ability and plant growth rate were generally lower in sand than in clay sediment at any given level of WER.

## 5 SUMMARY

In summary, increasing WER promoted the production of longer and thinner roots due to increasing oxygen availability, especially in plants grown in sand sediment, which subsequently enhanced nutrient (N and P) absorption and consequently the growth of the two submerged macrophytes. Submerged macrophytes usually play vital roles in water purification, suppression of harmful algae blooms, and maintenance of ecosystem stability. Consequently, alterations in the growth patterns and morphological and physiological characteristics of submerged macrophytes may have far‐reaching implications for ecosystem functioning, which highlights the need for further study on WER variation and its effects.

## CONFLICT OF INTEREST

None declared.

## AUTHOR CONTRIBUTIONS

Y. P. designed the experiments. Y. P. wrote the manuscript. D.Y.Y., X.H.M., J.J.C., W.G., and X.J.L. performed all experiments. C.Q.D. and Z.H.W. analyzed the data.

## DATA ACCESSIBILITY

Data deposited in the figshare repository: https://doi.org/10.6084/m9.figshare.7231349.

6
