# Supplementary material for: Effects of water exchange rate on morphological and physiological characteristics of two submerged macrophytes from Erhai Lake
Source: Ecol Evol. 2018 Nov 20;8(24):12750–60. doi: 10.1002/ece3.4703 (PMC6308862; doi:10.1002/ece3.4703)
Supplement: Supplementary file 1 [file ECE3-8-12750-s001.pdf]

Running title: plant responses to water exchange rate

Effects of water exchange rate on morphological and physiological characteristics of two submerged macrophytes from Erhai Lake

Duan-yang Yuan[1], Xianghuai Meng[1], Chang-qun Duan[1], Zhi-Hong Wei[2], Wei Gao[1], Jun-jun Chang[1], Xing-ju Lv[2], Ying Pan[1,3*]

[1]School of Ecology and Environmental Sciences & Yunnan Key Laboratory for Plateau Mountain Ecology and Restoration of Degraded Environments, Yunnan University, Kunming, Yunnan, 650091, China,

[2]Research Center of Erhai Lake, Dali, Yunnan 671000, China,

[3]Department of Biology, Nanjing University, Nanjing, 210093, China,

[*]Corresponding author: E-mail: panying@ynu.edu.cn

Tel: +86 871 65033547; fax: +86 871 65033547

Supplementary Figure S1 Illustration showing the experimental design for a water-exchange-rate treatment.

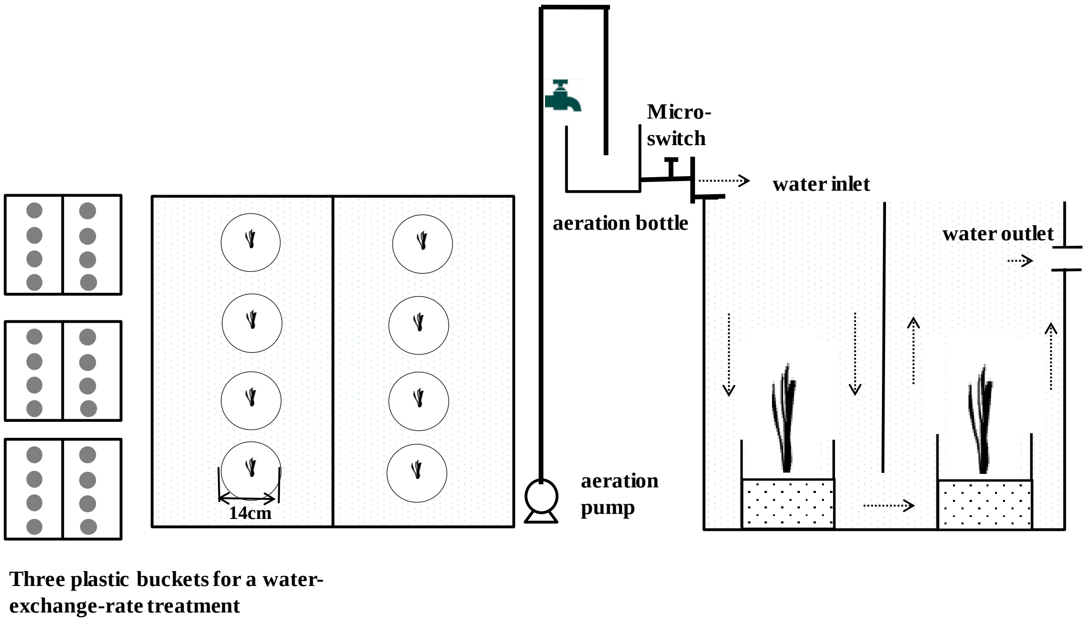

Supplementary Figure S2 Variations (mean ± 1 s. d., n = 3) in dissolved oxygen concentration on day 50 in both daytime and nighttime in plastic buckets with different water exchange rates in the experiment containing ramets. Different letters indicate significant differences among treatments. Multiple comparisons of means were performed using the Tukey test at the 0.05 significance level.

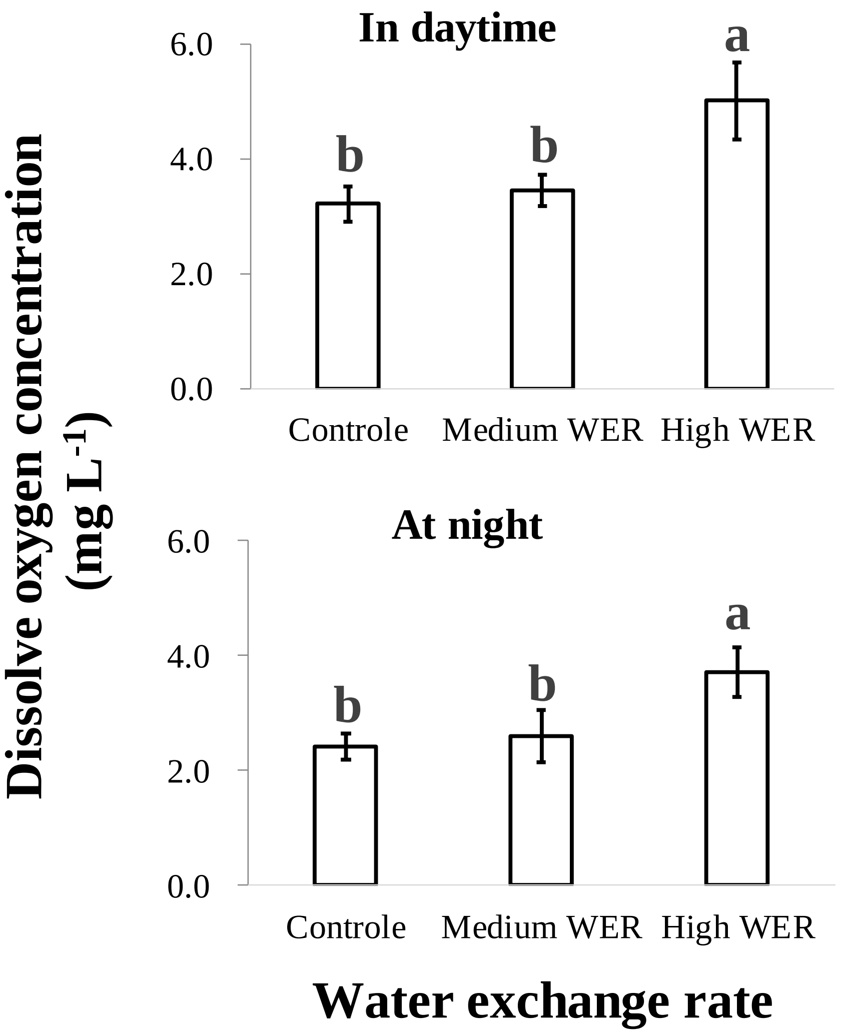

Supplementary Figure S3 Linear regressions between sediment oxidation-reduction potential and the root/above-ground biomass ratio in *Hydrilla verticillata* (A and C) and *Myriophyllum aquaticum* (B and D) in both daytime (A and B) and nighttime (C and D) at the end of the experiment containing ramets, under three levels of water exchange rate and two types of sediment.

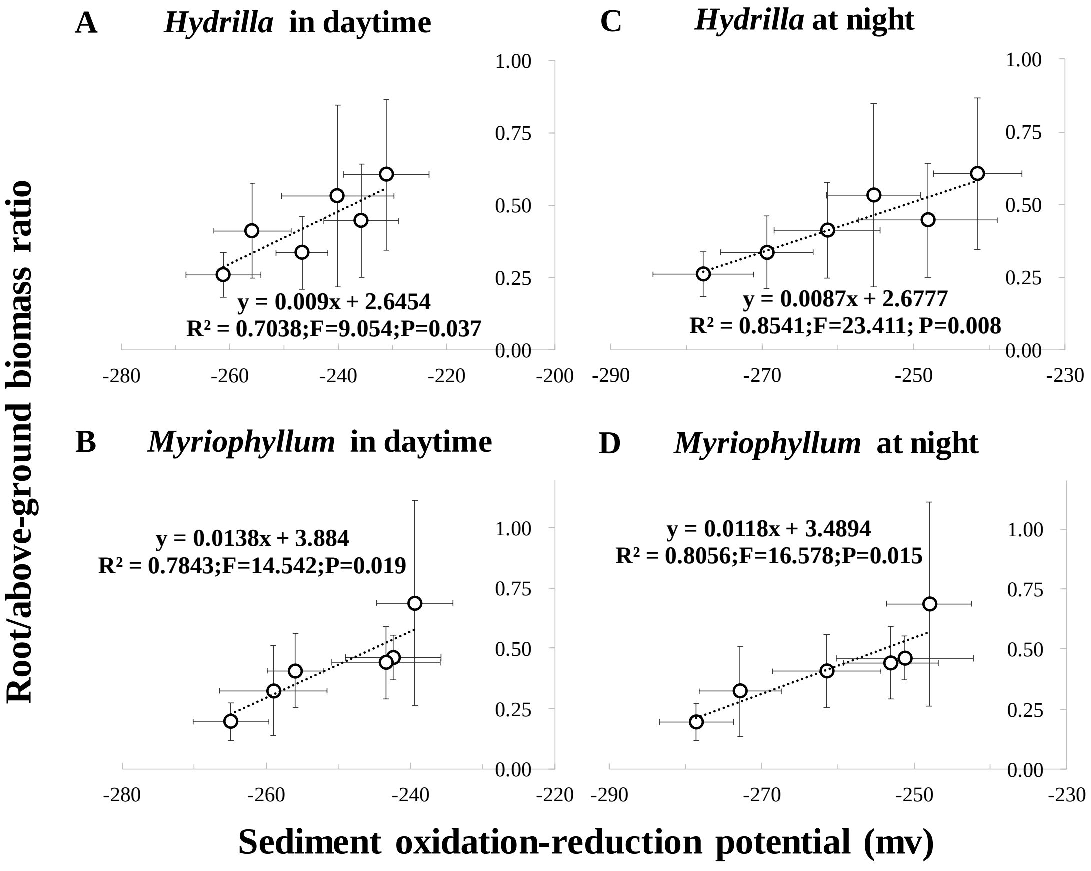

Supplementary Figure S4 Linear regressions between sediment oxidation-reduction potential and mean root length in *Hydrilla verticillata* (A and C) and *Myriophyllum aquaticum* (B and D) in both daytime (A and B) and nighttime (C and D) at the end of the experiment containing ramets, under three levels of water exchange rate and two types of sediment.

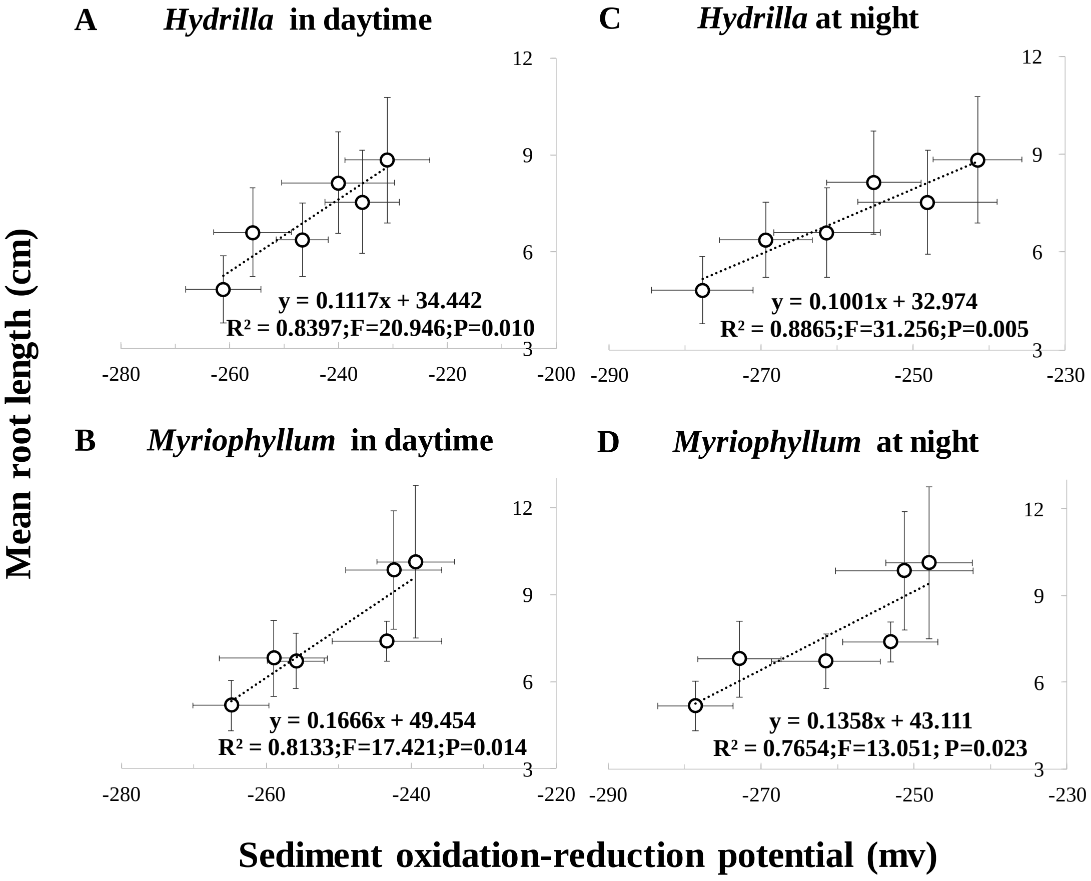

Supplementary Figure S5 Linear regressions between sediment oxidation-reduction potential and root diameter in *Hydrilla verticillata* (A and C) and *Myriophyllum aquaticum* (B and D) in both daytime (A and B) and nighttime (C and D) at the end of the experiment containing ramets, under three levels of water exchange rate and two types of sediment.

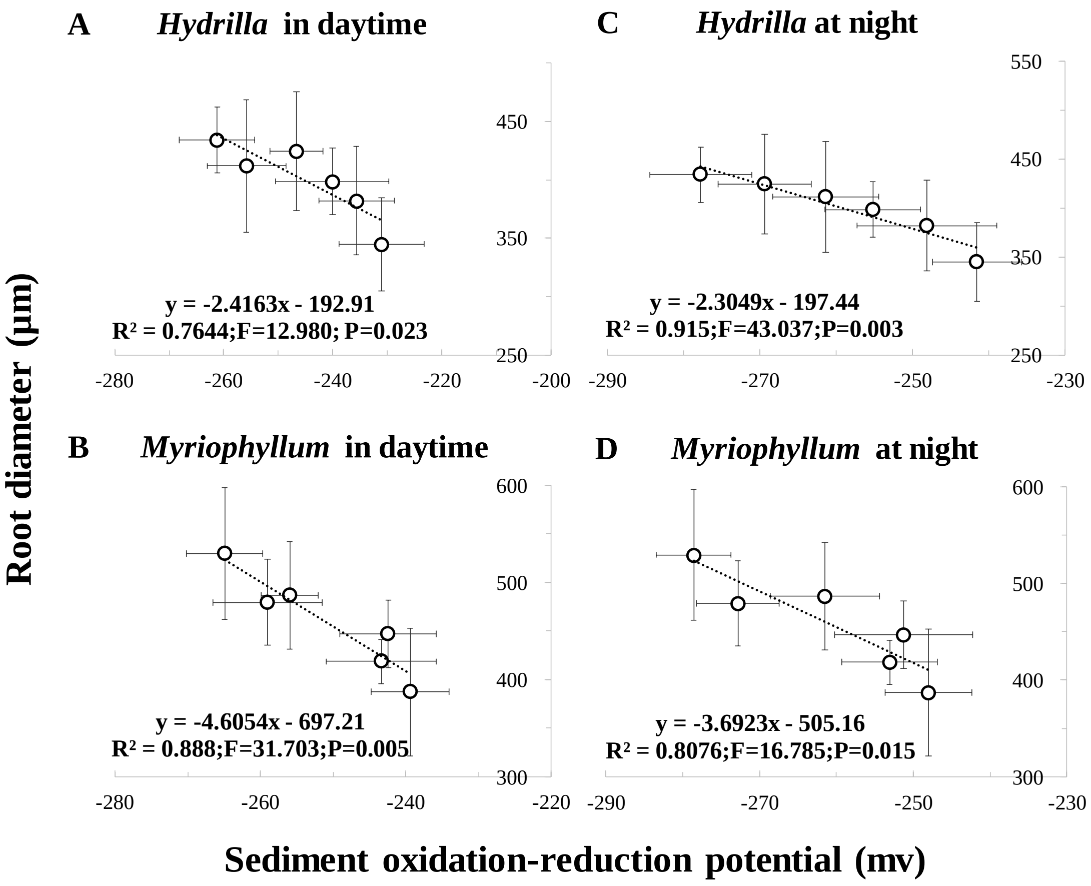

Supplementary Figure S6 Linear regressions between sediment oxidation-reduction potential and specific root length in *Hydrilla verticillata* (A and C) and *Myriophyllum aquaticum* (B and D) in both daytime (A and B) and nighttime (C and D) at the end of the experiment containing ramets, under three levels of water exchange rate and two types of sediment.

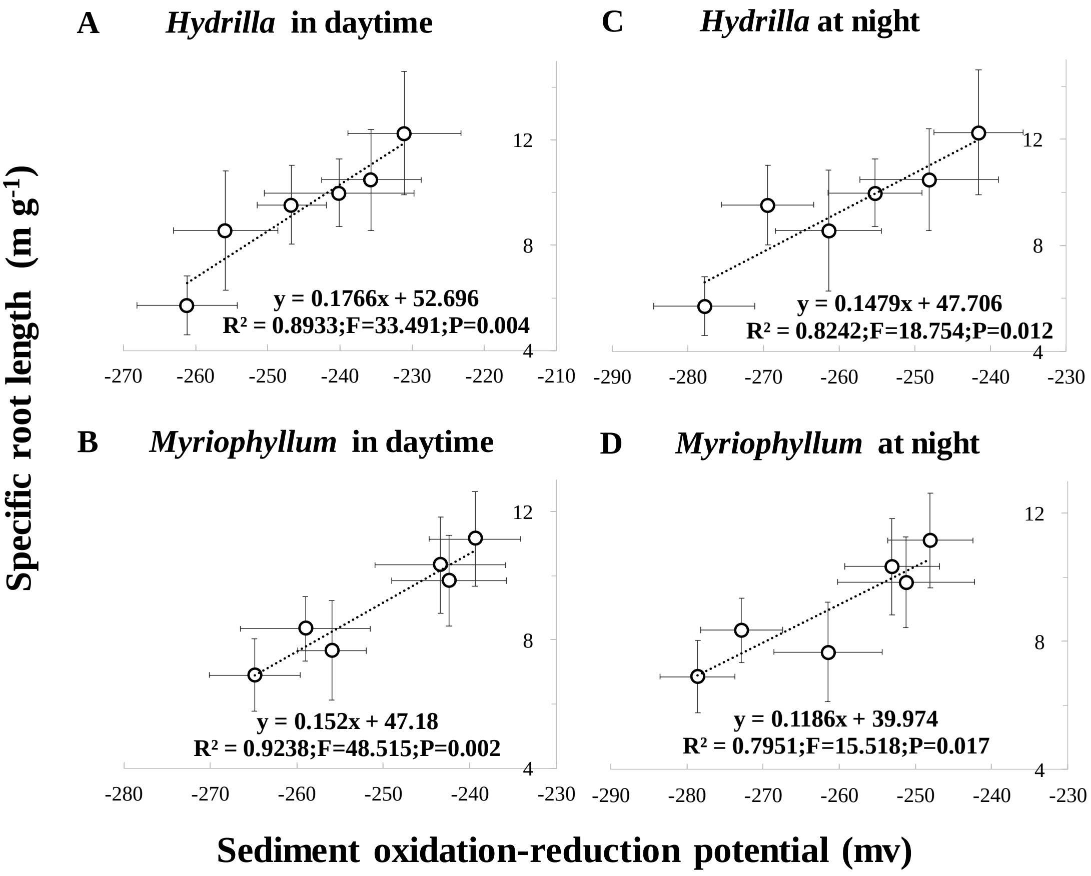

Supplementary Figure S7 Linear regressions between the concentration of plant nitrogen with the ratio of root biomass to above-ground biomass (root/above-ground biomass ratio, A and B), mean root length (C and D), root diameter (E and F) and specific root length (G and H), in *Hydrilla verticillata* and *Myriophyllum aquaticum* at the end of the experiment containing ramets, under three levels of water exchange rate and two types of sediment.

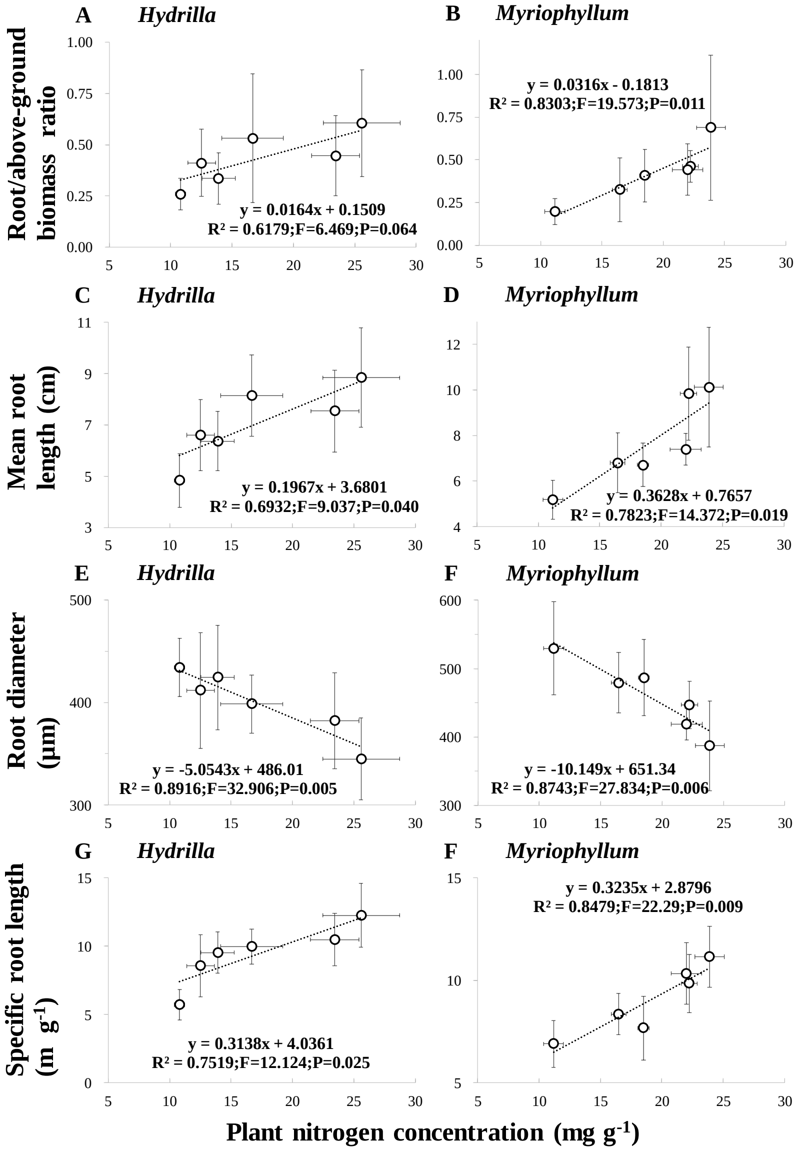

Supplementary Figure S8 Linear regressions between the concentration of plant phosphorus with the ratio of root biomass to above-ground biomass (root/above-ground biomass ratio, A and B), mean root length (C and D), root diameter (E and F) and specific root length (G and H), in *Hydrilla verticillata* and *Myriophyllum aquaticum*, under three levels of water exchange rate and two types of sediment.

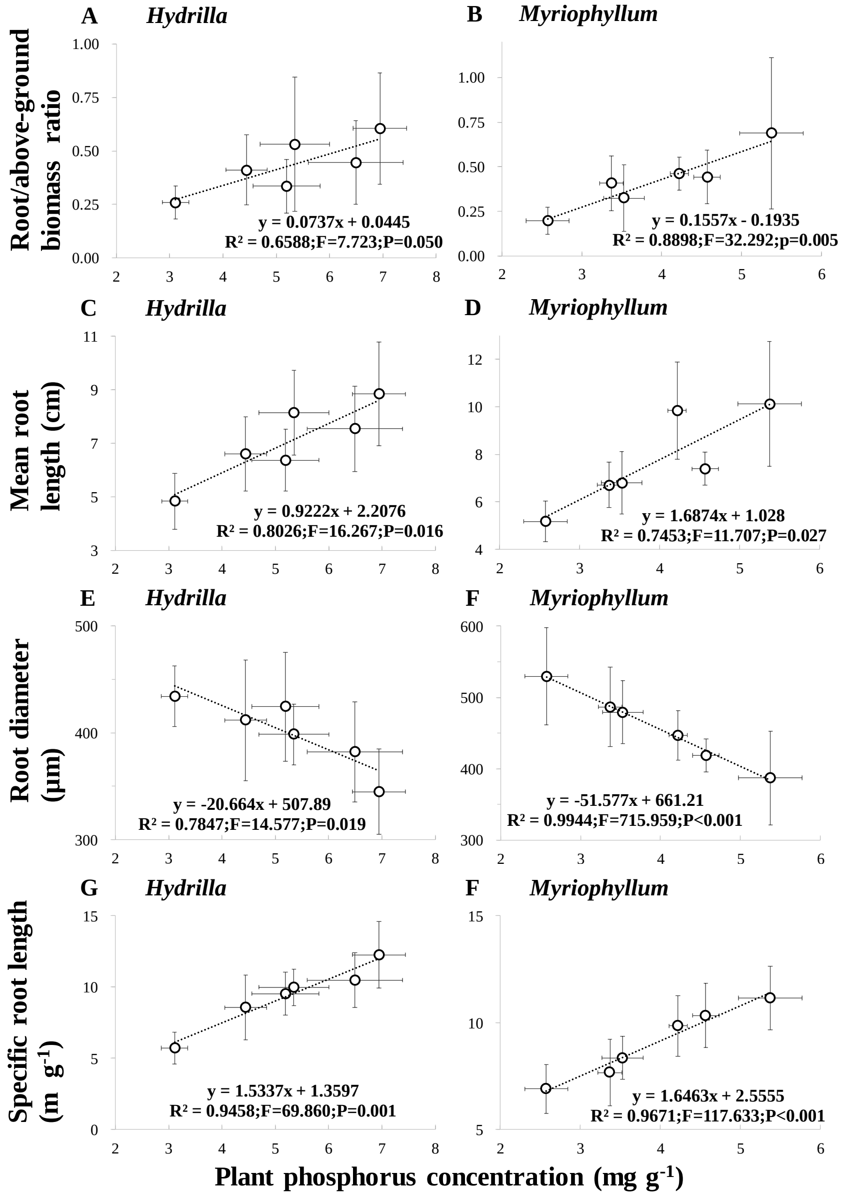

Supplementary Figure S9 Linear regressions between plant nutrients (nitrogen and phosphorus) with plant biomass accumulation (A-D) and relative growth rate (E-H) in *Hydrilla verticillate* and *Myriophyllum aquaticum*, respectively, under three levels of water exchange rates and two types of sediment.

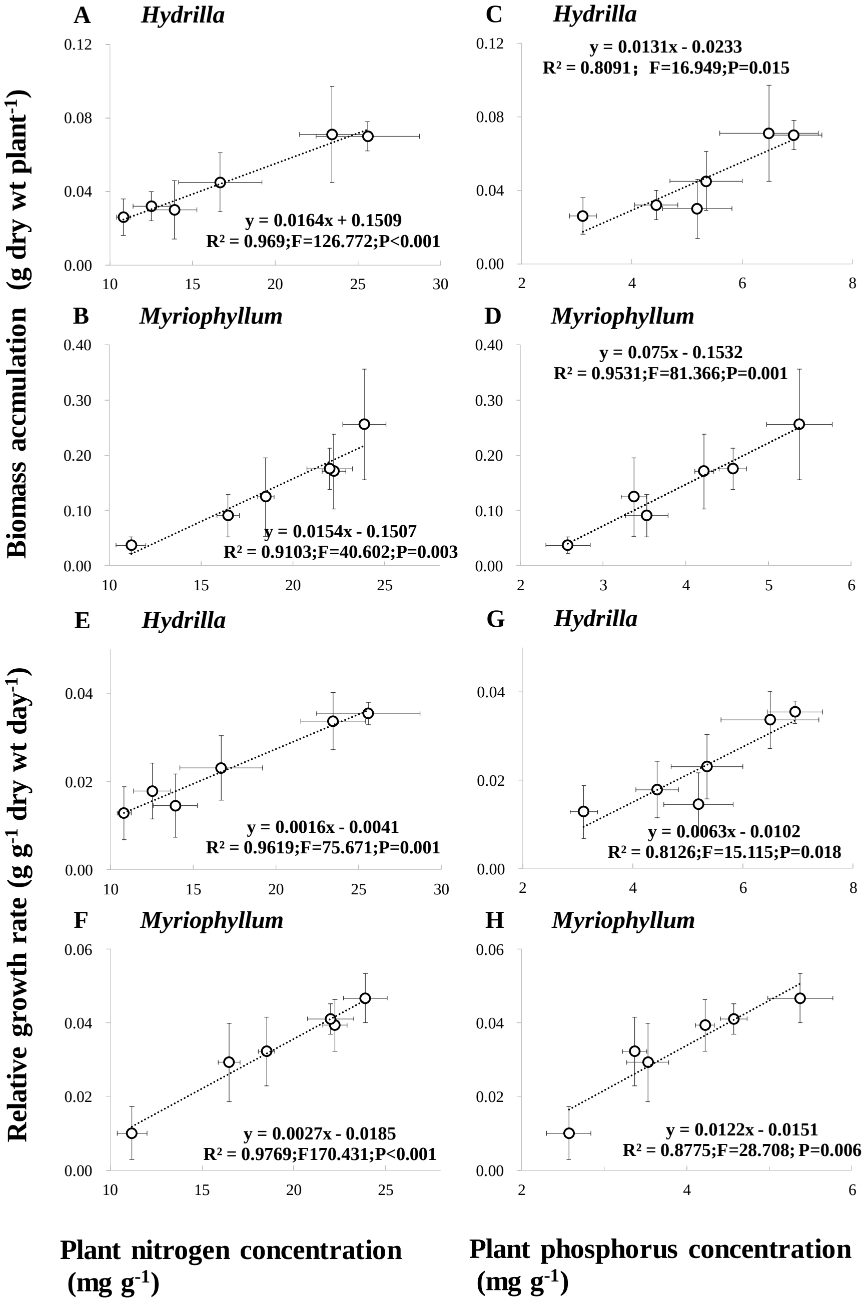

Supplementary Table S1 Variations in organic matter content and bulk density in two different sediment types at the initial of the experiments. Different letters indicate significant differences among treatments. Multiple comparisons of means were performed using the Tukey test at P = 0.05, following one-way ANOVAs.

Sediment type	*n*	Organic matter content ($g\ kg^{-1}$)	Bulk density ($g\ cm^{-3}$)
Clay	3	55.96 ± 6.80a	1.26 ± 0.02b
Sand	3	53.12 ± 9.18a	1.63 ± 0.04a

Supplementary Table S2 Variations (n = 3) in turbidity in treatments with different water-exchange rate (WER) in both daytime and nighttime during the 11-days-long experiment without ramets. Different letters indicate significant differences among treatments. Multiple comparisons of means were performed using the Tukey test at P = 0.05, following one-way ANOVAs.

	WER	n	Turbidity in daytime (NTU)	Turbidity in nighttime (NTU)
Day 2	Control	3	0.85±0.07a	0.86±0.12a
	Medium WER	3	0.84±0.08a	0.84±0.08a
	High WER	3	0.84±0.11a	0.83±0.09a
Day 4	Control	3	0.91±0.11a	0.92±0.08a
	Medium WER	3	0.90±0.11a	0.87±0.04a
	High WER	3	0.87±0.06a	0.87±0.06a
Day 6	Control	3	1.01±0.15a	0.99±0.14a
	Medium WER	3	0.99±0.09a	0.98±0.10a
	High WER	3	0.95±0.09a	0.92±0.08a
Day 8	Control	3	1.08±0.16a	1.06±0.13a
	Medium WER	3	1.01±0.13a	1.04±0.09a
	High WER	3	0.99±0.15a	0.96±0.12a
Day 10	Control	3	1.24±0.12a	1.21±0.13a
	Medium WER	3	1.08±0.11a	1.09±0.20a
	High WER	3	1.08±0.05a	1.03±0.13a

Supplementary Table S3 Variations (*n* = 3) in pH value in treatments with different water-exchange rate (WER) in both daytime and nighttime during the 11-days-long experiment without ramets. Different letters indicate significant differences among treatments. Multiple comparisons of means were performed using the Tukey test at P = 0.05, following one-way ANOVAs.

	WER	*n*	pH value in daytime	pH value in nighttime
Day 2	Control	3	8.65±0.09a	8.65±0.18a
	Medium WER	3	8.66±0.08a	8.66±0.18a
	High WER	3	8.65±0.17a	8.69±0.11a
Day 4	Control	3	8.69±0.11a	8.66±0.08a
	Medium WER	3	8.64±0.08a	8.71±0.11a
	High WER	3	8.64±0.10a	8.64±0.20a
Day 6	Control	3	8.70±0.12a	8.73±0.06a
	Medium WER	3	8.67±0.16a	8.63±0.23a
	High WER	3	8.63±0.17a	8.64±0.26a
Day 8	Control	3	8.73±0.28a	8.67±0.37a
	Medium WER	3	8.70±0.15a	8.59±0.19a
	High WER	3	8.58±0.16a	8.58±0.16a
Day 10	Control	3	8.75±0.17a	8.59±0.21a
	Medium WER	3	8.61±0.19a	8.55±0.15a
	High WER	3	8.54±0.14a	8.48±0.20a

Supplementary Table S4 Variations (*n* = 3) in dissolved carbon dioxide (DCD) concentration in treatments with different water-exchange rate (WER) in both daytime and nighttime during the 11-days-long experiment without ramets. Different letters indicate significant differences among treatments. Multiple comparisons of means were performed using the Tukey test at P = 0.05, following one-way ANOVAs.

	WER	*n*	DCD concentration in daytime (ppm)	DCD concentration in nighttime (ppm)
Day 2	Control	3	351.33±29.70a	348.65±26.48a
	Medium WER	3	363.67±30.89a	367.65±44.47a
	High WER	3	363.00±25.51a	365.43±46.13a
Day 4	Control	3	340.33±22.50a	352.60±20.21a
	Medium WER	3	352.67±38.42a	362.08±27.06a
	High WER	3	346.33±21.83a	359.29±33.48a
Day 6	Control	3	340.67±18.15a	350.21±39.99a
	Medium WER	3	364.67±5.69a	369.23±17.99a
	High WER	3	347.33±26.76a	356.35±28.60a
Day 8	Control	3	323.67±24.66a	341.25±17.89a
	Medium WER	3	368.33±21.03a	361.28±18.25a
	High WER	3	337.33±24.21a	345.19±21.64a
Day 10	Control	3	331.67±24.99a	324.26±11.23a
	Medium WER	3	363.00±12.77a	362.18±26.25a
	High WER	3	345.33±13.32a	332.76±20.27a

Supplementary Table S5 Variations (*n* = 3) in dissolve oxygen (DO) concentration in treatments with different water-exchange rate (WER) in both daytime and nighttime during the 11-days-long experiment without ramets. Different letters indicate significant differences among treatments. Multiple comparisons of means were performed using the Tukey test at P = 0.05, following one-way ANOVAs.

	WER	*n*	DO concentration in daytime (mg L^{-1})	DO concentration in nighttime (mg L^{-1})
Day 2	Control	3	4.30±0.17b	4.62±0.16b
	Medium WER	3	4.64±0.23ab	4.84±0.07ab
	High WER	3	4.87±0.15a	4.94±0.11a
Day 4	Control	3	4.95±0.28b	4.95±0.14b
	Medium WER	3	5.46±0.27ab	5.48±0.2ab
	High WER	3	5.65±0.27a	5.74±0.30a
Day 6	Control	3	5.07±0.16b	5.09±0.18b
	Medium WER	3	5.55±0.24ab	5.49±0.25ab
	High WER	3	5.95±0.19a	5.92±0.18a
Day 8	Control	3	5.37±0.15b	5.33±0.26b
	Medium WER	3	5.77±0.17ab	5.48±0.13b
	High WER	3	6.05±0.26a	6.03±0.22a
Day 10	Control	3	5.28±0.40b	5.28±0.20b
	Medium WER	3	5.73±0.14ab	5.63±0.22ab
	High WER	3	6.09±0.17a	5.99±0.13a

Supplementary Table S6 Variations (*n* = 3) in sediment oxidation-reduction potential (ORP) in treatments with different water-exchange rate (WER) in both daytime and nighttime at the end of the 11-days-long experiment without ramets. Different letters indicate significant differences among treatments. Multiple comparisons of means were performed using the Tukey test at P = 0.05, following one-way ANOVAs.

	WER	*n*	Sediment ORP in Daytime (mv)	Sediment ORP in nighttime (mv)
Clay	Control	3	-141.27±4.34b	-156.67±4.51b
	Medium WER	3	-97.70±1.31a	-111.03±1.35a
	High WER	3	-97.10±3.32a	-105.9±1.85a
Sand	Control	3	-265.77±2.70e	-270.20±3.02e
	Medium WER	3	-229.33±5.51d	-236.00±4.36d
	High WER	3	-199.10±2.09c	-203.93±1.01c

Supplementary Table S7 Variations (n = 3) in dissolve oxygen (DO) concentration in treatments with different water-exchange rate (WER) on days 10, 20, 30 and 40 in daytime in the experiment containing ramets. Different letters indicate significant differences among treatments. Multiple comparisons of means were performed using the Tukey test at P = 0.05, following one-way ANOVAs.

	n	DO 10 (mg L^{-1})	DO 20 (mg L^{-1})	DO 30 (mg L^{-1})	DO 40 (mg L^{-1})
Control	**3**	5.16±0.33b	5.05±0.24b	5.00±0.20b	4.33±0.43b
Medium	**3**	5.71±0.39ab	5.41±0.40ab	5.39±0.31b	4.60±0.43b
High WER	**3**	6.26±0.41a	6.20±0.35a	6.15±0.14a	5.93±0.17a

Supplementary Table S8 Summary of ANOVA results (F and P values) showing the effects of water exchange rate (WER, three levels) and experimental time (in daytime and at night) on water dissolve oxygen (DO) concentration (n = 3) on day 50 and sediment oxidation-reduction potential (ORP, n = 5) on day 51 in the experiment containing ramets, respectively.

	df	DO concentration (mg L^{-1})	Sediment ORP of *Hydrilla verticillata* (mv)	Sediment ORP of *Myriophyllum aquaticum* (mv)
WER	2	24.195***	40.461***	50.849***
Time (*T*)	1	25.054***	37.814***	27.781***
WER × *T*	2	0.630^{ns}	1.711^{ns}	1.048^{ns}
